# Social exercise interventions for children who have complex developmental needs: A systematic review

**DOI:** 10.1177/13674935231190984

**Published:** 2023-07-20

**Authors:** Kate Freire, Rod Pope, Isabella Size, Kristen Andrews, Emma Fitz-Gerald, Tricia Bowman

**Affiliations:** 1 Three Rivers Department of Rural Health, 1109 Charles Sturt University, Albury, NSW, Australia; 2 School of Allied Health, Exercise and Sports Sciences, 1109 Charles Sturt University, Albury, NSW, Australia; 3 Child and Family Services, 259655 Royal Far West, Manly, NSW, Australia; 4 Division of Library Services, 1109 Charles Sturt University, Albury, NSW, Australia

**Keywords:** child, exercise, developmental disabilities, social interaction

## Abstract

Exercise interventions are identified as effective treatments for children not meeting developmental milestones. This systematic review synthesizes research regarding exercise interventions that involved social participatory elements, for children with complex developmental needs. Academic Search Complete, CINAHL, Emcare, Proquest Theses and Dissertations, MEDLINE, and Google Scholar were searched systematically for relevant studies. Peer-reviewed studies meeting the review aim and published between 2000 and 2021 in English, were included. Methodological quality of 49 eligible studies (47 controlled trials, two mixed methods, total of 2355 participants) was appraised using the Mixed Methods Appraisal Tool. Narrative synthesis identified two groups of studies: Group 1 incorporated intentional social participatory elements; Group 2 likely involved incidental social participation. Most studies were of moderate to low methodological quality. Few measured impacts of interventions upon total physical activity levels. Short-term improvements in physical outcomes – particularly motor skills – were most frequently reported and were the main benefit of social exercise interventions for children with complex developmental needs, for which evidence exists. Further rigorous, longitudinal research is needed to assess social, psychological, and executive function outcomes of social exercise interventions in this population. Such interventions should incorporate booster sessions to provide children with greater opportunity to *meet* developmental milestones.

## Introduction

Participation in regular physical activity (PA) helps maintain physical and mental wellbeing (Lubans et al., 2016). Physical activity with social participatory elements such as active play during school recess, team sports or family-based PA, provides children with opportunities to practice prosocial skills including communication skills, taking turns, and conflict resolution and gaining these skills may contribute to children’s enjoyment and participation in PA (O’Connor et al., 2014). The World Health Organisation recommends that children accrue a minimum of 60 min of moderate-to-vigorous PA (MVPA) per day but globally many children are not meeting those guidelines (Tremblay, 2014). Cross-sectional studies have found that children’s levels of PA are positively correlated to their ability to perform fundamental motor skills (FMS), whereby those more proficient in FMS have higher levels of PA; and those with less proficiency participate in less PA (Lubans et al., 2010). This association is not surprising as FMS provide foundational skills, such as locomotor, object-control, and stability skills, to participate in many different types of PA, including sport-related activities (Lubans et al., 2010). It is important for children to gain proficiency in these skills as early as possible as the relationship between ability and participation in PA may strengthen over time (Stodden et al., 2008). However, directionality of this relationship and its many probable mediators aren’t fully understood (Barnett et al., 2011). Children who have complex developmental needs are a particular group who are at greater risk of inactivity and reduced FMS (Ferguson et al., 2015).

Children with complex developmental needs may have a single identified developmental disorder, or in a substantial number of children, they may experience concurrent developmental conditions (Kadesjo and Gillberg, 1999). Conditions may impact their physical ability, for example, Developmental Coordination Disorder (DCD) which is a broad diagnosis encompassing motor difficulties from immaturity and developmental delay (McWhirter et al., 2022). Other conditions may also impact them behaviourally and socially including autism spectrum disorder (ASD), which impacts social and communication skills, repetitive behaviours, and narrow interests across a wide spectrum (Bremer et al., 2015); and attention deficit hyperactivity disorder (ADHD), characterised by inattention, hyperactivity, and impulsive behaviours (O’Connor et al., 2014). Some children who are not meeting their developmental milestones have no formal diagnosis (Weitzman and Wegner, 2015). Management of these complex developmental presentations are important to reduce the risk of secondary health and psychosocial complications (Piek et al., 2008). Physical activity has been identified as an effective treatment, due to the physical, socio-emotional, behavioural, and cognitive benefits that it provides (Den Heijer et al., 2017). Exercise is PA, that is, structured and planned (Dasso, 2018). Recent systematic reviews have focussed upon using exercise to manage ADHD, (Cerrillo-Urbina et al., 2015; Den Heijer et al., 2017) rather than contextual elements of exercise interventions.

## Aim

To identify, critically appraise, and synthesise findings from published studies of exercise interventions that involved social participatory elements, in children with complex developmental needs.

## Methods

The protocol for this systematic review was registered with PROSPERO (CRD42020188327). Methods and findings are reported using the PRISMA 2020 statement (Page et al., 2021) as a guide.

### Identification of studies

Systematic searches for relevant articles were completed in February 2021 via the following databases and search engine: Academic Search Complete, Cumulative Index to Nursing and Allied Health Literature, Emcare, Proquest Theses and Dissertations, MEDLINE, and Google Scholar. Key search words and subject headings, related to PA, exercise and the relevant population, in conjunction with appropriate Boolean operators, were identified by reviewers. Search terms included: PA, exercise, sports, recreation, developmental vulnerability, motor skills, child, intervention, and program. Specific search strategies, adapted for each database by the librarian, are reported in supplementary material.

#### Inclusion/exclusion criteria

All identified citations, from searches, were uploaded to EndNote X9 (Clarivate Analytics, 2013) and duplicates removed. Title and abstract of each remaining report were screened against inclusion and exclusion criteria by one reviewer and clearly ineligible articles were removed.

Inclusion criteria included studies which included children aged 2–18 years who were identified as not meeting their developmental milestones, studies that evaluated an exercise intervention which included social participatory elements, research published between 2000 and 2021 in English language, and peer reviewed published research including quantitative, qualitative, and mixed methods studies, theses.

Exclusion criteria included were studies that did not evaluate an exercise intervention or described an exercise intervention with no social elements, and review articles, conference proceedings and posters; non-peer-reviewed research, including grey literature and opinion papers.

Reports were retained, and full text retrieved, if they appeared to be potentially eligible or if there was any uncertainty regarding eligibility, and all decisions were recorded. Full texts of these articles were then assessed for eligibility by two reviewers, independently. Disagreements were resolved by a third reviewer and excluded reports were removed from further consideration, with reasons for exclusion recorded. Reference lists of all reports selected for inclusion, based on eligibility criteria, were screened to identify additional eligible studies.

### Assessment of methodological quality

Methodological quality of included studies was appraised using the Mixed Methods Appraisal Tool (MMAT) (Hong et al., 2018). This tool allows assessment of five different types of study using a single tool. Two reviewers independently scored each study against five quality criteria and disagreements were resolved by a third reviewer. As recommended by Hong et al. (2018), no studies were excluded from this study because of their methodological quality assessment results.

### Data extraction and synthesis

Information extracted from each study included: authors, year of publication, study design, setting, participant characteristics (gender, age, developmental needs), intervention description, comparator (if any), sample size, study duration, analysis method, main outcome measure(s), secondary outcome measure(s), quantitative results, and qualitative findings. Extracted data were documented in standardised tables which had been piloted by all reviewers using a small number of studies, to ensure consistency between reviewers in data extraction. Data extraction was completed by two review authors independently and then combined into one table. Differences were resolved through discussion and consensus.

Data were synthesised using a narrative approach (Popay et al., 2006). Meta-analysis was not conducted because the studies were not sufficiently homogenous for a meaningful analysis due to diversity of participants, interventions, and outcomes, as well as lack of sufficient data. Findings from individual studies were tabulated and sorted, guided by the results of the MMAT methodological quality appraisals.

## Results

### Identification and selection of studies

**Figure 1.**
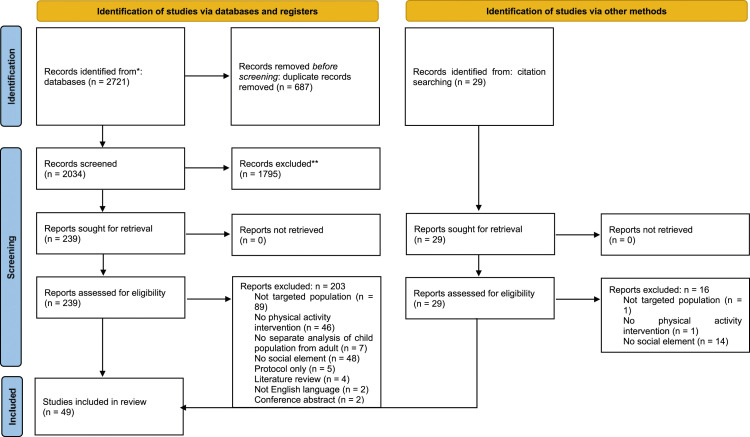
PRISMA flow diagram (Page et al., 2021) depicting literature search, screening and selection processes.

Figure 1 summarises results of identification, screening, and selection of studies in a PRISMA flow diagram (Page et al., 2021). After duplicate removal, 2063 studies remained for screening. Title and abstract screening resulted in 1795 papers being excluded and 268 full texts of studies were obtained for review. Full text reviewing excluded 219 studies, leaving 49 eligible studies.

### Characteristics of included studies

Characteristics of included studies are collated in Table 1. Forty-two (85%) included studies were from the second decade of publication covered by the review (2011–2021). Included studies originated from 17 different countries, with nearly a quarter of studies from USA (*n* = 11, 22%). Twenty (41%) studies took place in a school or education setting and three (6%) studies were conducted at a pre-school. Health (*n* = 10, 20%) and university settings (*n* = 9, 18%) were also frequently described. Study setting was not described by six (12%) studies.

**Table 1.** Characteristics of included studies.

Authors, date, country	Setting	Participants
Ahmed and Mohamed, 2011, Saudi Arabia	3 schools for children with disability	84 children with ADHD, aged 11–16 years. 2 equal groups IG and CG (NI)
Angeli et al., 2019, USA	Outpatient paediatric medical centre	19 children & young adults with physical disabilities, aged 7–24 years. Diverse movement disabilities with CP 70%
Apache, 2005, USA	2 pre-school classes	2 classes of 28 pre-schoolers with developmental delays, aged 3–6 years. Each class participated in both intervention
Bo et al., 2019, China	NR	9 boys with diagnosis of ASD, aged 7–12 years
Bremer et al., 2015, Canada	Child treatment centre	8 children with ASD, aged 4 years. IG *n* = 5, waitlist CG *n* = 3
Bremer and Lloyd, 2016, Canada	Elementary school	5 children with ASD, aged 3–7 years
Caçola et al., 2016, USA	University Recreation Centre	24 children with DCD, aged 7–12 years. 2 groups: IGA *n* = 11, IGB *n* = 13
Chang et al., 2014, China	2 elementary schools	27 children with ADHD, aged 5–10 years. 2 groups: IG *n* = 14, waitlist CG *n* = 13
Chou and Huang, 2017, China	Dance studio	49 children with ADHD, aged 8–12 years. 2 groups: IG *n* = 24, CG *n* = 25 (NI)
Da Silva et al., 2020, Brazil	School	20 children with ADHD, aged 11–14 years. 2 groups: IG *n* = 18, CG *n* = 15
Dickinson and Place, 2014, UK	3 classes for children with ASD	100 children with ASD, aged 5–15 years. 2 groups: IG *n* = 50, CG *n* = 50. CG continued with standard school-based PE program.
Ekins et al., 2019, Germany	School for children with ID	15 children with an ID or developmental disability such as ASD, aged 11–15 years. 2 groups: IG *n* = 10, PE group *n* = 5
Favazza et al., 2013, USA	50 pre-school classes from 26 schools	233 children with intellectual disabilities, aged 3–5 years. 2 groups: IG *n* = 113, CG *n* = 120
Ferguson et al., 2013, South Africa	3 primary schools	46 children with DCD, aged 6–10 years. 2 groups: NTT program *n* = 27, Wii program *n* = 19
Ferguson et al., 2015, South Africa	Primary school	41 children aged 6–10 years old. 22 children with DCD, 19 TD children
Fong et al., 2012, China	Child assessment centres and hospitals	Children with DCD & TD children aged 6–9 years. 3 groups: DCD-Taekwondo group *n* = 16, DCD-CG (NI) *n* = 13, TD-CG (NI) *n* = 10
Golubović et al., 2014, Serbia	Sports school	36 boys aged 5–7 years 2 groups, Group 1: Boys with hyperactivity *n* = 18, Group 2: NT boys *n* = 18
Hung and Pang, 2010, China	Child assessment centre	Children with DCD aged 6–9 years. 2 groups: Group setting *n* = 12, individual setting *N* = 11
Işık and Zorba, 2020, Turkey	Special education classes in schools	50 youth with mild to moderate ID, aged 12 –16 years. 2 groups: IG *n* = 25, CG *n* = 25 (NI)
Kane and Staples, 2016, Canada	Pediatric rehab program	10 boys with gross motor difficulties, aged 5–7 years
Ketcheson et al., 2017, USA	Autism health centre	20 children with ASD, aged 4–6 years. 2 groups: IG *n* = 11, CG *n* = 9, (NI)
Ketcheson et al., 2021, USA	University gymnasium	24 children with intellectual and developmental disabilities, aged 4–13 years
Kosari et al., 2013, Iran	Department of Education	20 males with ADHD, aged 8–9 years. 2 groups: IG *n* = 10, CG *n* = 10 (NI)
Lau et al., 2020, China	School	194 children with mild ID, aged 10–15 years old. 2 groups: IG *n* = 121; CG *n* = 73 (NI)
Lee et al., 2015, Republic of Korea	University hospital	12 boys with ADHD, aged 7–9 years. 2 groups: IG *n* = 6, CG *n* = 6 (NI)
Memarmoghaddam et al., 2016, Iran	Sixth education district in Tehran	36 boys with ADHD, aged 7–11 years. 2 groups: IG *n* = 19, CG (NI) *n* = 17
Najafabadi et al., 2018, Iran	Therapeutic clinic	26 children with ASD, aged 5–12 years. 2 groups: IG *n* = 12, CG *n* = 14 (NI)
Ninot et al., 2005, France	School	32 females with ID, aged 13 and 17 years. 4 groups (*n* = 8): ID only swimming group, open swimming group, PE group, CG (NI)
O’Connor et al., 2014, USA	NR	98 children with ADHD, aged 5–8 years. 2 groups: STP program *n* = 52, CG *n* = 46 (NI)
Oreskovic et al., 2020, USA	University campus, participants homes	12 participants with ASD, aged 6–10 years
Őzer et al., 2012, Turkey	Special education and secondary school	Male youth with ID (*n* = 38) and TD youth (*n* = 38), aged 13–15 years. 2 groups: IG (youth with ID *n* = 23, TD youth *n* = 23). CG (NI)
Pan et al., 2016, Taiwan	University table tennis centre	Boys with ADHD aged 6–12 years. Group 1 *n* = 16, Group 2 *n* = 16. Group 1 received intervention, Group 2 was CG (NI), then reversed
Pan et al., 2017a, Taiwan	University gymnasium	24 boys with ADHD and 24 TD boys aged 7 to 14 years. IG boys with ADHD *n* = 12, CG1 boys with ADHD, *n* = 12, CG2 TD boys *n* = 24
Pan et al., 2017b, Taiwan	University multipurpose room	Boys with ASD aged 6–12 years. Group I *n* = 11, group II *n* = 11. Group 1 received intervention, Group 2 was CG (NI), then reversed
Peens et al., 2008, South Africa	9 primary schools	58 children with DCD, aged 7–9 years. 4 groups: Motor intervention, self-concept, psycho-motor intervention, CG (NI)
Pless, 2001 study 2, Sweden	Child health centre	Children with motor difficulties consistent with DCD, aged 5–6 years. 2 groups: IG *n* = 17, CG *n* = 20 (NI)
Rafie et al., 2017, Iran	NR	20 adolescents with ASD, aged 9–12 years. 2 groups: IG *n* = 10, CG *n* = 10 (NI)
Regaieg et al., 2020, Tunisia	NR	28 children with Down Syndrome (13 girls and 15 boys; aged 6–10 years. 2 groups: IG *n* = 13, CG *n* = 15 (no intervention)
Rubin et al., 2018, USA	Participants homes	111 youth with Prader–Willi syndrome (PWS) or obesity, aged 8–16 years. IG – children with PWS *n* = 34, children with obesity *n* = 43. Waitlist CG – children with PWS *n* = 11; children with obesity *n* = 23.
Siu and Lo, 2020, China	NR	14 children with ADHD, aged 7–9 years, 25 parents
Smith et al., 2013, USA	School	14 children who scored ≥4 on the disruptive behavior disorders rating scale, aged 5–8 years
Smith, 2015, Canada	Children’s centre	13 girls with ASD, aged 8–11 years
Sullivan, 2017, USA	Elementary & middle school	Children with ID, ASD, ADHD, or LD aged 7–15 years. 2 groups, IG *n* = 14, CG (NI) *n* = 14
Tsai, 2009, Taiwan	School	43 children aged 9–10 years old. Children with DCD *n* = 27, TD children *n*=16. 3 groups IG children with DCD, CG with DCD, TD-CG
Tsai et al., 2012, Taiwan	NR	Children with DCD & TD children aged 9–10 years 3 groups: DCD soccer group *n* = 16), DCD-CG (NI) *n* = 14, TD-CG group (NI) *n* = 21
Tsiotra, 2010, study 3, Greece	4 elementary schools	20 children with DCD and 48 TD, aged 7–14 years. DCD-IG *n* = 10, TD-IG *n* = 22, DCD-CG *n* = 10, TD-CG *n* = 26. 2 CG PE curriculum
Valentini et al., 2017, Brazil	University campus	Children with disabilities (*n* = 18) (CP, ADHD, ASD), and TD children (*n* = 46) aged 6–10 years. 2 groups: Mastery climate *n* = 32, exercise play *n* = 32
Verret et al., 2012, Canada	Hospital and school	21 children with ADHD, aged 7–12 years. 2 groups: IG *n* = 10, CG *n* = 11
Ziereis and Jansen, 2015, Germany	University Institute of Sport	39 children with ADHD, aged 7–12 years. 3 groups: IG1 *n* = 12, IG2 *n* = 11, CG *n* = 16 (NI)

*Notes*: CG: control group; CP: cerebral palsy; ID: intellectual disability; IG: intervention group; NI: no intervention; NR: not reported; NTT: Neuromotor Task Training; TD: typically developing.

Numbers of participants in intervention groups ranged from five to one hundred and twenty-one participants. Nearly three quarters of studies (*n* = 36, 73%) included less than 20 participants in their intervention group. Fourteen studies (29%) included pre-schoolers (aged 3–5 years), forty-three (88%) included children aged six to 12 years and twelve (24%) included children aged 13 years and older. Children with a range of developmental conditions were included in the studies, including ADHD (*n* = 14, 29%), ASD (*n* = 10, 21%), DCD (*n* = 9, 18%), mixed developmental conditions (*n* = 5, 10%), and intellectual disability (ID) (*n* = 5, 10%).

### Quality screening

Of the included studies, twenty (41%) were quantitative randomised controlled trials (RCT), twenty-seven (55%) were quantitative non-randomised controlled trials (NRCT) and two were mixed method studies (supplementary material). Methodological quality of studies varied, with 11 (22%) of studies deemed high quality (scoring 5 on MMAT), 20 (41%) medium quality (scoring 3 or four on MMAT), and 18 (37%) low quality (scoring ≤2 on MMAT). Nearly three quarters (*n* = 13, 72%) of those evaluated as low quality used a RCT methodology. Quality assessment of RCT studies found that 15 (75%) did not randomise the population adequately, in 14 (70%) outcome assessors were not blinded to type of interventions, and eight (40%) indicated poor participant adherence to assigned intervention. A third (*n* = 9, 33%) of NRCT studies were deemed high quality. Among all NRCT studies, information on conduct of intervention or exposure was not provided by 12 (44%) and nine (33%) did not account for confounders in the design and analysis. Neither mixed method study met criterion of achieving specific quality standards for each method utilised in their design.

### Synthesis

#### Overview

Two broad groups of studies were identified. Twenty-four (49%) studies incorporated social participation with other participants as an integral part of exercise interventions (Table 2) and formed Group One. Examples of exercise interventions in these studies included team sports or collaborative activity such as: catching practice. Only three (12.5%) of these studies were deemed high quality using MMAT (supplementary material). Another twenty-five (51%) studies were identified where social participation between participants was *likely* to have been an incidental outcome of the group context (Table 3) and formed Group Two. Examples of exercise interventions in this second group of studies included exercise involving exercise stations, individual exercise within a group such as yoga or free play, and online group games. Nearly a third of studies in Group Two (*n* = 8, 32%) were deemed high quality using the MMAT (supplementary material). A summary is provided as supplementary material of intervention schedules from both groups of studies and outcome measures used in the included studies (across physical, social, psychological, executive function and other domains). Within group analysis found wide diversity in outcome measures employed. For clarity, the next two sections focus primarily on synthesising findings from studies deemed to be of high quality, based on MMAT results, with only brief commentary provided on findings from lower quality studies, where warranted.

**Table 2.** Group one: studies which incorporated social participation with other participants as an integral part of their group intervention.

Authors, date	Intervention	Focus of outcome measures					Results	Author reported	Author reported
		PHY	SOC	PSY	EF	OTH		Facilitators	Barriers
Caçola et al., 2016	Content: Program A – task-orientated activities in large group involving motor skills training, collaboration, and cooperation. Program B – direct goal program, 3 small groups addressed the motor goals chosen by the children.	✓	✓	✓	✗	✗	Significant improvement in MABC total score for both groups. Higher anxiety and lower levels of enjoyment found in program A participants compared to decreased anxiety in program B participants post intervention. Program A parents reported an improvement in rate of function and a decrease in peer problems of their child. Program B parents noted higher control of movement in their children.	Group aspect provides fun for children.	The variety of difficulties children with DCD have and maturity may limit engagement in group interventions.
	Social: Both groups completed activities individually, in pairs and in a group.								
	Schedule: 60 min/week × 10 weeks.								
Ekins et al., 2019	Content: IG: dynamic movement and drumming on exercise ball, including strength based, team exercises. PE sessions: Running, jumping, throwing.	✓	✓	✗	✗	✗	No significant improvement in any outcome measure.	NR	NR
	Social: IG included turn-taking, sharing, taking cues from each other and team exercises.								
	Schedule: Duration of sessions NR. IG x2 drums alive session and x2 PE sessions per week, × 7 weeks. CG × 3 PE sessions per week × 7 weeks.								
Favazza et al., 2013	Content: IG program included motor skills - walking, running, balance, jumping, trapping, catching, and motor games, cool down motor song. Home component- suggestions for home activity and complete home record.	✓	✓	✗	✗	✗	IG significant improvement compared to CG on peabody developmental motor scale. Improvement in kindergarten readiness skills and social/play skills.	NR	Behavioural issues, lack of adults to maintain adult to child ratio and difficulty with motor tasks.
	Social: Classroom groups and HEP.								
	Schedule: 30 min x3/week × 8 weeks.								
Işık and Zorba, 2020	Content: IG hemsball training program focused towards gaining skills for the game which requires accurate throwing skills in single leg stance and catching skills.	✓	✗	✗	✗	✗	Significant improvement of IG compared to CG in BOT, with larger improvement found in participants with moderate ID.	Hemsball can be played indoors or outdoors.	NR
	Social: Training completed in pairs.								
	Schedule: 60 min x3/week × 12 weeks.								
Kane and Staples, 2016	Content: Gross motor skills program. Program included children and their parents, who took part in all activities except warm up. Aerobic and strength activities, practice of skills related to child’s individual goals.	✓	✗	✗	✗	✓	No significant improvement in TGMD scores. No significant improvement in MVPA. No change in sedentary activity. Nine children reported improved performance of one of their goals.	Breakdown and adapt activities to promote success. Parent involvement facilitated carry-over of intervention to home and community.	Children need help to find activities that are suited to their abilities and interests.
	Social: Training in a group with parents as their training partner.								
	Schedule: 120 min x3/week for 7 sessions.								
Lee et al., 2015	Content: IG jump rope and swiss ball exercise program. Intensity of swiss ball exercises and rope-jumping gradually increased.	✓	✗	✗	✗	✗	IG - significant improvement in cardiorespiratory endurance, muscle strength, muscle endurance, flexibility, and epinephrine level.	NR	NR
	Social: Included couple and group rope jumping.								
	Schedule: 60 min x3/week × 12 weeks.								
Memarmoghaddam et al., 2016	Content: IG program to improve executive function. Goal directed exercises, for example, keeping the ball on the racket while walking, ball games.	✗	✗	✗	✓	✗	Significant improvement in IG in executive function. Significant difference between the IG and CG in inhibition.	NR	NR
	Social: Individual and team activities.								
	Schedule: 90 min x3/week × 8 weeks.								
Najafabadi et al., 2018	Content: IG motor and behavioural skills program. Activities: Aerobic dance, running games, soccer, basketball, frisbee.	✓	✓	✗	✗	✗	Total BOT score IG NR. Significant improvement in IG balance (static and dynamic) and bilateral coordination. IG significant improvement in both subscales of autism rating scale.	NR	NR
	Social: Team sports and team activities.								
	Schedule: 40 min × 3/week for 12 weeks.								
O’Connor et al., 2014	Content: IG - intensive behaviour modification camp. Skill drills to practice their sports abilities. Team sports: Soccer, tee-ball, softball, and basketball. Practice of fundamental skills, game strategies and rules.	✓	✓	✗	✗	✗	IG significant in BOT scores. Improvement in sport knowledge & performance for both soccer & tee ball than CG. IG group significant improvements in trapping, dribbling penalties, and catching accuracy than CG. No differences between groups on kicking, throwing, or hitting accuracy. Parent ratings found that IG group improved significantly more than CG on specific sports skills and good sportsmanship.	NR	NR
	Social: Team sports and collaborative activities.								
	Schedule: 9 h/day × 8 weeks. Swimming instruction duration NR. Skill drill × 60 min/day. Team sport × 120 min/day.								
Őzer et al., 2012	Content: IG soccer program. Followed the special olympics football coaching guide (2004): Drills, soccer skills, team tactics and match tactics. Soccer tournament at the end which parents were invited to attend.	✗	✓	✓	✗	✗	Significant improvement in friendship activity scale in IG, no change in CG. IG - significant improvement in CBCL in students with ID, no change in TD youth. CG no change found. IG – no significant improvement in the adjective checklist.	NR	NR
	Social: Team sports.								
	Schedule: 90 min x3/week × 8 weeks.								
Pan et al., 2016	Content: IG motor skill program, social behaviours & cognitive functions. Including: Motor skills practice, executive function training using table tennis exercise, group games.	✓	✓	✗	✓	✗	No significant improvement in any outcome measure.	Parents reported children enjoyed the sessions.	NR
	Social: Group activities, 2:1 or 1:1 child-to-instructor ratios.								
	Schedule: 70 min x2/week × 12 weeks.								
Pan et al., 2017a	Content: IG simulated developmental horse-riding program - riding machine, physical fitness training: Running, basketball, exercise stations, ball games.	✓	✗	✗	✗	✗	Significant improvements in the BOT, cardiovascular endurance, and flexibility of the IG.	Attendance was incentivised with money and a start chart.	NR
	Social: Team sport and activities.								
	Schedule: 90 min/week × 12 weeks.								
Pan et al., 2017b	Content: Both groups completed table tennis program. Sessions included: Motor skills, motor training aimed at executive function, games.	✓	✗	✗	✓	✗	Significant improvement in total BOT scores for both groups. Group 1 maintained their improvements after 2^nd^ 12-week period. Significant improvement in half of the indices of Wisconsin card sorting test.	NR	NR
	Social: Activities in pairs focused on social interaction and sportsmanship development.								
	Schedule: 70 min x2/week × 12 weeks.								
Pless, 2001 study 2	Content: IG motor skills program. Practice of functional skills: Balance, ball-playing and gross motor skills.	✓	✗	✗	✗	✗	No significant improvement IG test scores.	NR	NR
	Social: At end of session played a game together.								
	Schedule: x1/week for 10 weeks. Duration of sessions NR.								
Rafie et al., 2017	Content: IG program - motor activities, games, and sports. Focus upon body awareness, motor planning, motor balancing skills, coordination, visual-motor skills.	✓	✗	✗	✗	✗	Significant improvement in BOTMP scores of IG compared to CG.	NR	NR
	Social: Team sports. Parents invited to watch IG.								
	Schedule: 45 min x3/week × 10 weeks.								
Regaieg et al., 2020	Content: IG adapted PE program – football, long jump and sprint adapted exercises. Based on games situations: Activities and games that focused on taking turns and tag.	✓	✗	✗	✗	✗	IG significant improvement in TGMD scores.	NR	NR
	Social: Team sports and activities.								
	Schedule: 60 min x2/week × 10 weeks.								
Rubin et al., 2018	Content: IG playground activity using balls, hoops, hurdles, and cones. Active video games.	✓	✗	✗	✗	✗	IG no significant improvement MVPA, total PA levels and sensory organisation test scores. Significant improvement in following BOT subscales: Upper limb coordination, balance, strength.	NR	NR
	Social: Home based program completed with guardian.								
	Schedule: Duration increased from 25 to 45 min over 24 weeks, x2/week playground and x2/week Nintendo Wii.								
Siu and Lo, 2020	Content: Rugby skills programr: Skills, drills, cooperative games. Family rugby in 3–4 subgroups.	✗	✓	✗	✗	✓	No significant improvement in any outcome measure. No details provided on how researchers used child participants’ drawings.	Children enjoyed asking parents to join in. Sun morning, in central location, to facilitate attendance.	NR
	Social: Skills training and group game with parents.								
	Schedule: 90 min x1/week × 6 weeks.								
Smith, 2015	Content: Multi-sport skills camp focused on gross motor skills including balance, running, jumping, galloping, hopping, kicking, bouncing, catching; throwing, striking.	✓	✓	✓	✗	✓	Significant improvements in TGMD scores, physical self-perceptions, sport and athletic competence, total score of children and youth physical self perception profile. No change PA levels. Significant correlations between motor skills & physical self-perceptions, & between physical self-perceptions & social skills.	Majority of camp counsellors were female which facilitated participation and role modelling.	NR
	Social: Sport-skills training and team sports.								
	Schedule: 7 h/day × 1 week.								
Sullivan, 2017	Content: IG gross motor program: Body skills & locomotor skills, ball skills & locomotor, play.	✗	✗	✗	✓	✗	Significant improvement in IG NEPSY-II subtests compared to CG. No difference between groups on BRIEF rating scale.	NR	NR
	Social: Team sport and collaborative play.								
	Schedule: 20 min x5/week × 4 weeks.								
Tsai et al., 2012	Content: Soccer program: Lower-extremity exercises, soccer-specific technical skills, short intermittent sprint; comprehensive practice, a soccer match.	✓	✗	✗	✓	✗	Significant improvement in MABC score, response accuracy and reaction time (RT) for DCD-soccer group post training.	NR	NR
	Social: Team sport.								
	Schedule: 50 min x5/week × 10 weeks.								
Tsiotra, 2010, study 3	Content: DCD-IG & TD-IG motor skills and exercise program. Skills: Throwing at a target. Exercise: Running, skipping and flexibility.	✓	✗	✓	✗	✗	Significant improvement BOT scores in DCD-IG and TD-IG. No significant difference between IG and CG before and after intervention in the Children’s self perception of adequacy in and predilection for PA scale and PA participation questionnaire	NR	NR
	Social: Individual and group training including games for ball handling.								
	Schedule: 45 min x3/week × 8 weeks.								
Verret et al., 2012	Content: IG motor skill program. Physical activities: Basketball, soccer, exercise stations, tag, and ball games to maintain moderate to vigorous intensity during session.	✓	✓	✗	✓	✗	No significant improvement in TGMD scores in IG. Significant improvement for some subscales of the child behaviour checklist for IG. Significant improvement level of information processing of IG.	NR	NR
	Social: Team sport and collaborative games.								
	Schedule: 45 min x3/week × 10 weeks.								
Ziereis and Jansen, 2015	Content: 2 physical programs. IG1: Ball skills, balance, catching, throwing, balance training, tennis; juggling; beach volleyball, handball. IG2: Sports without a specific focus- relay races; swimming; wrestling games; climbing; orienteering; gymnastics; track and field.	✓	✗	✗	✓	✗	IG1 and IG2 significant improvement in MABC scores compared to CG. No significant difference between IG1 and IG2. IG1 and IG2 significant improvement in working memory scores compared to CG. No significant difference between IG1 and IG2.	NR	NR
	Social: Sport and collaborative activities.								
	Schedule: 60 min/week × 12 weeks.								

*Notes*: BOT: Bruininks–Oseretsky test of motor proficiency; CBCL: child behaviour checklist; CG: control group; EF: executive function; HEP: home exercise program; ID: intellectual disability; IG: intervention group; MABC: movement assessment battery for children; MVPA: moderate to vigorous physical activity; NR: not reported; OTH: other; PA: physical activity; PE: physical education; PHY: physical; PSY: psychological; SOC: social; TD: typically developing; TGMD: test of gross motor development.

**Table 3.** Group two: studies where social participation between participants were likely an incidental part of the group context.

Authors, date	Intervention	Focus of outcome measures					Results	Author reported	Author reported
		PHY	SOC	PSY	EF	OTH		Facilitators	Barriers
Ahmed and Mohamed, 2011	Content: IG aerobic exercise program. Moderate-intensity exercises: Upper limb, lower limb, trunk, and neck exercises, running. Week 6–10: HEP.	✓	✓	✗	✓	✗	Significant improvement in IG motor skills, attention and academic and classroom behaviour. No significant differences between groups in task orientation and emotional and oppositional behaviour. Results of HEP NR.	NR	NR
	Social: walk together as cool down. HEP with parents.								
	Schedule: x3/week for 10 weeks. Week 1–4: 40 min sessions, week 5–10: 50 min. Weekend walk 30 min.								
Angeli et al., 2019	Content: Community running program. Progressed walk-run intervals until 5 km distance reached. Encouraged to walk x2/week and rest x3/week. 5 km race at program end.	✗	✗	✓	✗	✗	Significant improvements in self-perception domains of scholastic competence, athletic ability, and physical appearance. No significant improvement in self-perception of social competence and behavioural conduct.	NR	NR
	Social: Running in small groups (3–4 people including volunteer).								
	Schedule: x2/week × 10 weeks. Duration of sessions NR.								
Apache, 2005	Content: Activity-based program vs direct instruction program. Programs included 3 units: Locomotor unit, object control unit. Mixed unit: golf, and obstacle courses.	✓	✗	✗	✗	✗	Significant improvement in TGMD for participants of the activity-based program compared to the direct instruction program.	Teachers in an activity-based program that teachers play alongside the students for greater interaction with the students.	NR
	Social: Training completed in classroom groups. Activity-based intervention had greater instructor involvement and interaction with students.								
	Schedule: 30 min x3/week × 15 weeks.								
Bo et al., 2019	Content: Locomotor and ball skills program based upon skills in TGMD. Focus on one locomotor skill and one ball skill each day, classroom pivotal response Teaching. Was used in instruction process.	✓	✗	✗	✗	✗	Significant improvements on TGMD scores. Participants with greater social impairment showed greater improvements on locomotor and overall TGMD in comparison to those with lower social impairment score.	NR	NR
	Social: 1:1 child-to-instructor ratios. Free play, breaks, yoga, cool downs, and instructions as a group.								
	Schedule: 3.5 h x5/week × 2 weeks.								
Bremer et al., 2015	Content: Fundamental movement skills program. Session: Review of previously learned skill, direct instruction and practice of new skill, obstacle course, free play.	✓	✓	✗	✗	✗	Significant improvement in PDMS scores. Increased time spent in appropriate play but no significant improvement in Vineland adaptive behaviour scale, social skills improvement system, and behaviour during free play. No difference between the groups found.	Low-cost intervention implemented in a community setting. Group dynamics worked well in small group size while still providing opportunities for social interactions.	NR
	Social: 2:1 and 1:1 child-to-instructor ratios for training. Group obstacle courses and free play.								
	Schedule: 60 min/week × 12 weeks or 60 min x2/week × 6 weeks.								
Bremer and Lloyd, 2016	Content: Motor skills program. Review of previously skills, instruction, practice of new skills. Object control: Catching, throwing, kicking, and locomotor: Jumping, running, galloping. Obstacle course, ride on bikes.	✓	✓	✗	✗	✗	Results not analysed statistically. Improvement in TGMD and social skills improvement system scores.	NR	Some children had difficulty focussing on task due to sensory overload of gym environment.
	Social: 1:1 child: Instructor, completed in a group.								
	Schedule: 45 min x3/week × 6 weeks. Two blocks separated by 6 weeks.								
Chang et al., 2014	Content: IG aquatic exercise program. Moderate-intensity water aerobic exercise, perceptual-motor water exercise including games for coordination, balance, and power.	✓	✗	✗	✓	✗	IG – significant improvement in target throwing and bead moving subscales of basic motor ability test and in accuracy of go-no-go task. Waitlist CG results post intervention NR.	NR	NR
	Social: Group aquatic program.								
	Schedule: 90 min x2/week × 8 weeks.								
Chou and Huang, 2017	Content: IG yoga program. Yoga activity focused on concentration, balance, breath, and body awareness.	✗	✗	✗	✓	✗	Significant improvements in visual pursuit test and determination test of IG compared to CG.	NR	NR
	Social: Yoga exercises performed in a group.								
	Schedule: 40 min x2/week × 8 weeks.								
Da Silva et al., 2020	Content: IG swimming program. Swimming exercises: Leg propulsion, arms, breathing, crawl.	✓	✗	✓	✓	✗	IG: Significant improvement lower limb laterality, flexibility, abdominal resistance, depression, stress, cognitive flexibility, and selective attention. Comparison with CG NR.	NR	NR
	Social: Group swimming training								
	Schedule: 45 min x2/week × 8 weeks.								
Dickinson and Place, 2014	Content: IG computer-based activity program - Nintendo Wii and the software package Mario and sonics at the Olympics. PE: standard school PE program.	✓	✓	✗	✗	✗	IG significant improvement on all tests (shuttle run, long jump and partial curl up tests) other than flexibility compared to CG. No significant differences in family functioning between both groups.	Staff reported that children enjoyed the intervention.	NR
	Social: 2–4 participants and pairs.								
	Schedule: Computer-based activity program 15 min x3/week, PE: 30–45 min x2/week, school weeks for 1 year.								
Ferguson et al., 2013	Content: Neuromotor task training (NTT) program - guided by groups motor control level and goal of playing outdoor games. Included: Obstacle courses, running, jumping, throwing activities. Practise of components of selected games. Wii program: Gaming on Wii fit balance board.	✓	✗	✗	✗	✗	Significantly greater improvement in NTT group scores in motor performance, functional strength & cardiorespiratory fitness compared to Wii group. No improvements in isometric strength were seen in either group.	NR	NR
	Social: Small groups with individual workstations.								
	Schedule: NTT – 45–60 min x2/week x9 weeks. Wii – 30 min x3/week x6 weeks.								
Ferguson et al., 2015	Content: DCD and TD groups. Program based upon guidelines from WHO school health initiative and university curriculum. Encouraged play and PA, supported with PE lessons. Details of intervention NR.	✓	✗	✗	✗	✗	Significant improvement in both DCD and TD groups in motor skills.	NR	NR
	Social: Classroom groups.								
	Schedule: Duration and frequency NR, x9 weeks.								
Fong et al., 2012	Content: IG group – children with DCD. Taekwondo training. Punching, blocking horseback riding stance, kicking in fighting stance, stretching.	✓	✗	✗	✗	✗	IG: Significant improvement in sensory organisation and unilateral stance tests (UST).	NR	NR
	Social: Group training group and HEP.								
	Schedule: 60 min/week × 12 weeks 60 min/day HEP.								
Golubović et al., 2014	Content: Physical activity program. No details of description, type, intensity of intervention.	✓	✗	✗	✗	✗	No significant improvement in any outcome measure.	NR	NR
	Social: Group training.								
	Schedule: 60 min x3/week × 6 months.								
Hung and Pang, 2010	Content: Motor skill program. Functional tasks, agility, balance, core stability & movement coordination exercises.	✓	✗	✗	✗	✓	Significant improvement in MABC score for both groups. No significant difference between the 2 groups in MABC score and home exercise compliance. No significant difference in parent satisfaction of program.	Comparison by children motivating them to engage. A stronger sense of competence from demonstrating skills in front of peers.	NR
	Social: 2 groups - comparison between group training (4–6 participants) and individual setting. HEP both groups.								
	Schedule: 45 min/week × 8 weeks 20 min/day HEP.								
Ketcheson et al., 2017	Content: IG weekly rotation between object control and locomotion program. Classroom pivotal response Teaching manual was used to guide instruction.	✓	✓	✗	✗	✗	Significant improvement in IG TGMD scores post intervention. No significant difference in accelerometer PA levels. Playground observation of peer engagement: IG spent significantly less time in solitary play (CG not assessed).	NR	NR
	Social: Group instruction, instructor to participant ratio 1:1. IG given opportunities to interact during free play.								
	Schedule: 4 h x5/week × 8 weeks.								
Ketcheson et al., 2021	Content: Locomotor and ball skill program based on TGMD.	✓	✗	✗	✗	✗	No significant improvement in TGMD scores. Change in locomotor skill showed a moderate significant correlation with MVPA.	NR	The diversity of skills in children with IDD.
	Social: Small and large groups, instructor child ratio 1:1.								
	Schedule: 60 min/week x10 weeks.								
Kosari et al., 2013	Content: IG gross motor program. Motor displacement activities, manipulative motor skills.	✓	✗	✗	✗	✗	Significant improvement in BOT scores in IG.	NR	NR
	Social: Group intervention but no details on overt social activities.								
	Schedule: 45 min x18 sessions. Duration and frequency NR.								
Ninot et al., 2005	Content: 4 groups of adolescents with ID: Group 1: Swim training and swimming meets for adolescents with ID only. Group 2: Swim training and open swimming meets. Group 3: PE group: PA sessions, content NR. Group 4CG.	✓	✗	✓	✗	✗	Significant improvement in timed swim for groups 1, 2, & 3 with no differences between groups. No change in perceived general self-worth for the 4 groups. Group 2 significantly lower perceived athletic competence despite the increase in athletic performance.	NR	NR
	Social: Group training.								
	Schedule: 120 min/week × 32 months of training and 12 competitive meets. PE group NR.								
Oreskovic et al., 2020	Content: Walking program. Personalized daytime walking route for each participant.	✓	✗	✗	✗	✗	No significant improvement in any outcome measure.	NR	Family availability and commitment to the program.
	Social: Encouraged to complete with another family member but NR.								
	Schedule: 30 min or more daytime walk x3+/week x3 months.								
Peens et al., 2008	Content: 4 groups of children with DCD. Group 1: Motor program only. Group 2: Self-concept program only. Group 3 motor and self-concept programs. Group 4: CG. Motor program: Rolling, skipping, hopping, jumping, fine motor co-ordination and eye control activity.	✓	✗	✓	✗	✗	Groups 1 & 3 significant improvements in MABC. Group 2 no significant improvement in MABC. Group 2 & 3 significant improvement in total self-concept. No significant differences between groups in levels of anxiety.	Most of the activities could be practised individually.	The fine motor and eye control activities required special equipment and individual instruction.
	Self-concept program: 45 min x1/week for 8 weeks (physical, emotional, thinking, behaviour, and social self).								
	Social: Group training.								
	Schedule: 30 min x2/week × 8 weeks.								
Smith et al., 2013	Content: IG before school MVPA program. Aerobic running activity. Rotate through 4 stations focused on object control, skipping, running, hopping, crab walking.	✓	✓	✗	✓	✗	Significant improvement in BOT, motor timing task, shape school, and some subscales of the Pittsburgh Modified Conners Teaching rating scale in IG. No change with behaviours of not speaking nicely, intentional aggression, not following adult directions.	School bus transport to the program. Attendance certificates given regularly during program. Attendance award on the last day of the program.	Children sitting down when tired was managed by small reward system for those children who kept moving.
	Social: Rotating through exercise stations in small groups.								
	Schedule: 26 min × school days × 8 weeks.								
Tsai, 2009	Content: IG table tennis program. Serving, bouncing drills, smashing, driving, footwork, hitting back ball at the same speed as delivered. Program progressed in complexity.	✓	✗	✗	✓	✗	Significant improvement in MABC and inhibitory control of IG test scores compared to CG with DCD.	NR	NR
	Social: Group training completed with instructor.								
	Schedule: 50 min x3/week × 10 weeks.								
Valentini et al., 2017	Content: Mastery climate (MC) or exercise play (EP) program. Progression through 7–10 stations with 4–5 children per station.	✓	✓	✗	✗	✗	MC group significant improvement TGMD & significantly higher recall of verbal cues compared to EP group. No significant improvements found in the EP group.	NR	Children switched to skills they were more proficient in, limiting their practice time of the poorer skills.
	Social: Rotation through different stations in small group.								
	Schedule: 60 min × 2/week × 14 weeks.								
Lau et al., 2020	Content: IG Xbox sport season series 1 and 2. Included 6 team based and individual sports including boxing, bowling, volleyball, baseball.	✓	✗	✗	✗	✗	No significant improvement in any outcome measure.	Children preferred adventure games, but those games had less sport specific skills.	Bored of playing same game and reduced engagement due to difficulty with rackets and bats. Behaviour issues when competing. Difficulty following instructions.
	Social: Computer game played in pairs.								
	Schedule: 30 min x2/week × 12 weeks.								

*Notes*: BOT: Bruininks–Oseretsky test of motor proficiency; CG: control group; DCD: developmental coordination disorder; EF: executive function; HEP: home exercise program; ID: intellectual disability; IG: intervention group; MABC: movement assessment battery for children; MVPA: moderate to vigorous physical activity; NR: not reported; OTH: other; PA: physical activity; PDMS: Peabody developmental motor scales; PHY: physical outcome measures; PSY: psychological; SCQ: social communication questionnaire; SOC: social outcome measures; TD: typically developing; TGMD: test of gross motor development.

#### Group one: Studies which incorporated social participation as an integral part of exercise intervention

High quality studies which incorporated social participation as an integral part of exercise intervention described interventions that included motor skills training or skills training for specific collaborative sports, targeting children aged 5–14 years (*n* = 3, 12.5%). Two studies (O’Connor et al., 2014; Pan et al., 2017a) utilised the Bruininks–Oseretsky Test of Motor Proficiency (BOT-2) to assess participants’ motor skills, while Smith (2015) used The Test of Gross Motor Development (TGMD-2). Pan et al. (2017a) found significant improvement in BOT-2 scores, cardiovascular endurance, and flexibility post intervention. Their intervention included a simulated developmental horse-riding program, ball games and exercise stations; and finished with cool down activities focused on ‘prosocial behaviour and engagement’ (Pan et al., 2017a: 786). Pan et al. (2017a) only assessed physical outcome measures, despite their goal of providing an intervention that was suitable for children with ADHD and which included social elements. O’Connor et al. (2014) evaluated effect of sports training as part of an intensive behavioural modification program for children with ADHD and found significant improvements in motor and sports skills (performance and knowledge of game rules). They asked parents to rate their child’s improvement in ‘sports skills’ and ‘good sportsmanship’ using a Likert scale (O’Connor et al., 2014: 1010) and found that parents of children in the intervention group reported significantly larger improvement than parents of children in the no-intervention control group.

Smith (2015) used outcome measures assessing four domains to evaluate a one-week intensive, multi-sport, skills camp for girls with ASD (*n* = 13). They found significant improvement in TGMD-2 scores but no improvement in total PA levels immediately post program or at 8 weeks post-program. Improvements found in social and psychological domains, using questionnaires that were completed by both children (Children’s Self-Perceptions of Adequacy in and Predilection for Physical Activity) and guardians (The Social Skills Improvement System (SSIS), Vineland adaptive behaviour scale (VABS-2)), were not statistically significant. Children also completed the Children and Youth Self Perception Profile and reported significant improvements in sub-domains of physical self-perceptions, sport/athletic competence, and social skills. Significant correlations between motor skills and physical self-perceptions were also found, suggesting that as a child’s motor skills improved, their self-efficacy for PA and sport competence also increased.

Synthesis of findings from lower quality studies from Group One, listed in Table 2 (*n* = 21, 87.5%), identified that only two studies measured PA levels and found no significant change in PA levels post intervention (Kane and Staples, 2016; Rubin et al., 2018). Kane and Staples (2016) reported a non-significant improvement of 10-min of MVPA per day, but their small sample size (*n* = 10) is likely to have contributed to the null finding. Sixteen (76%) lower quality studies assessed motor skills and eleven (69%) of them found significant improvement in at least one aspect of participants’ motor skills. No discernible pattern was identified in social, psychological, or executive function outcome measures. However, it is worth noting the wide variation in measures and focus upon physical outcomes, as only nine (37.5%) studies assessed social outcomes, seven (29%) studies executive function outcomes, and four (17%) studies psychological outcomes.

#### Group two: Studies where social participation was likely to have been an incidental part of group context

High quality studies in Group Two, in which social participation was likely to have been an incidental part of group context, described exercise interventions that included motor skills training (*n* = 4, 50%) and obstacle courses (*n* = 3, 37.5%). Six (76%) high-quality studies in this group focused on only one domain of their participants’ development; specifically, four (50%) focused on physical outcomes, one (12.5%) on psychological outcomes, and one (12.5%) on executive function outcomes. Three (60%) of the five studies in Group Two that assessed motor skills utilised the Movement Assessment Battery for Children-2 and significant improvements in motor skills were found in all five studies. Three (37.5%) studies utilised other physical outcome measures, including Fong et al. (2012), who found significant improvements in sensory organisation and unilateral stance tests in 16 children with DCD following 3 months of Taekwondo training. No high-quality studies in Group Two assessed total PA levels post intervention.

Bremer et al. (2015) used parent questionnaires to assess social skills (SSIS) and adaptive behaviours (VABS-2), and behavioural video of free play, which was analysed by researchers; they found non-significant improvements across these measures in their sample of eight children with ASD. Angeli et al. (2019) used The Self Perception Profile for Children to assess self-concept following a 10-week community running program for children and young adults with disability (*n* = 19). Participants significantly improved their self-perception in domains of scholastic competence, athletic ability, and physical appearance but no significant improvements occurred in their self-perception of social competence and behavioural conduct. Chou and Huang (2017) found significant improvements in attention and discrimination function in children with ADHD following an 8-week yoga program.

Synthesis of findings from lower quality studies from Group Two (Table 3) (*n* = 17, 68%), identified that four (23.5%) studies assessed PA levels via accelerometer data, and none found significant improvements in PA levels post intervention. Most studies (*n* = 12, 70.5%) that investigated physical outcomes reported significant improvements in one or more outcome measure. Six (35%) studies examined social outcomes with mixed results; three (50%) reported significant improvements in at least one social outcome measure, including social and play skills. Three (18%) studies assessed a range of psychological attributes, with significant improvements found in self-concept, depression, and stress. Five (30%) studies investigated executive function post intervention, with significant improvements found for attention, cognitive flexibility, and response inhibition.

#### Adverse findings across both groups

Two (4%) lower quality studies reported adverse findings. Caçola et al. (2016) examined two interventions, both involving motor skills training; one was focussed on task orientated activities and another on goal directed activities. They found that anxiety increased, and enjoyment decreased in the task orientated group post intervention. The authors acknowledged that it was difficult to draw conclusions on whether this was an intervention effect because of confounding factors, in particular differences in group sizes. The task orientated group performed their intervention in one group (*n* = 11) whilst goal directed activities were conducted in three smaller groups of four-to-five participants.

Ninot et al. (2005) assessed social outcomes for adolescent females with ID, across four groups: a swimming group for participants with ID only, a mixed swimming group of children with ID and children who were typically developing (TD), a physical education group, and a control group (no intervention). They found no change in perceived self-worth across the four groups but children with ID in the mixed swimming group had a significantly lower self-perception of athletic competence post intervention, despite an increase in their performance.

#### Barriers and facilitators

Seven (28%) studies reported 11 facilitators, and three (12.5%) studies reported six barriers to implementing interventions which incorporated social participation with other participants as an integral part of exercise interventions (Group One; Table 2). Facilitators included incentivising attendance with money and a chart depicting participants’ attendance (Pan et al., 2017a); and parents facilitating carry-over from intervention to home environments (Kane and Staples, 2016). Enjoyment or fun was the most frequently mentioned facilitator (*n* = 3, 27%), whilst engagement barriers (*n* = 5, 83%) were the most frequently reported barriers. For example, Caçola et al. (2016) noted that the varying abilities of children with DCD limited engagement of some in group interventions.

Seven (28%) studies reported 11 facilitators, and seven (28%) studies reported 10 barriers to implementing interventions where social participation between participants were likely to be an incidental part of group context (Group Two; Table 3). Most of the identified facilitators related to engagement (*n* = 7, 64%), including: enjoyment of the intervention (Dickinson and Place, 2014) and the dynamics of small groups providing opportunities for social participation (Bremer et al. 2015). Barriers relating to engagement were most frequently reported (*n* = 5, 50%) and included children shifting to motor skills in which they had greater proficiency, which limited their practice time of motor skills in which they were less adept (Valentini et al., 2017).

## Discussion

The aim of this review was to synthesise current evidence relating to exercise interventions involving social participatory elements, for children who have complex developmental needs. Two broad groups of studies were identified: studies that incorporated social participation as an integral part of exercise intervention (Group One), and studies where social participation between participants were likely an incidental part of group context (Group Two). However, differences between these exercise intervention types in physical outcomes are currently unclear, as both groups of studies focussed predominantly upon physical outcomes, particularly motor skills. Intervention influence upon total PA levels is largely unknown, as only seven of the 49 included studies assessed total PA levels post intervention. Furthermore, the review identified aspects of social, psychological, and executive function domains that would benefit from additional investigation in future studies of group exercise interventions in this population. Finally, adverse findings, albeit identified in only two lower quality studies, highlight that group context may be an influential factor in a child’s experience of group exercise interventions.

This review found short-term improvements across many different physical outcomes following exercise interventions with social participatory elements, particularly in motor skills, but long-term implications of these improvements have not been studied. Future studies should consider longitudinal designs so that physical and psychosocial outcome persistence is investigated. It is also worth noting that although short-term improvements in motor skills were found, in many cases, these improvements did not allow children to *meet* their developmental motor milestones (Pan et al., 2017a, 2017b; Tsai et al., 2012). This finding suggests that future studies should not only consider longitudinal designs but also repeated intervention or booster sessions (Gearing et al., 2013). Booster sessions are now incorporated into behaviour change practices, acknowledging that enthusiasm and routines can fade over time, leading to relapses (Gearing et al., 2013). Gaining insights into both longitudinal outcome persistence and impacts of booster sessions would provide clinicians and families with more useful information from which to plan interventions.

All included studies utilised an exercise intervention, but only seven (14%) studies assessed short-term total PA levels post intervention. None of these studies found significant increases in total PA levels but their findings may have been impacted by small sample sizes. Thus, the influence of these interventions on total PA levels, both short- and long-term, is largely unknown and a significant gap in this area. Omission of total PA levels as an outcome measure is inconsistent with current understanding of a dynamic relationship between motor skills and PA (Barnett et al., 2011), and appears short-sighted considering potential health benefits and current low levels of total PA in this population (Lubans et al., 2010; O’Connor et al., 2014).

It seemed a reasonable hypothesis that high quality studies which integrated social elements into exercise interventions would observe greater improvements across multiple domains than those where social participation was incidental to the exercise. However, few studies assessed outcomes across multiple domains so limited insight was gained about impacts of group exercise interventions upon the social domain and other aspects of a child’s development. However, the review has identified several outcome areas that would benefit from further investigation, including sport skills and sportsmanship (O’Connor et al., 2014), social and play skills (Smith, 2015), adaptive behaviours (Bremer et al., 2015), self-concept (Angeli et al., 2019), self-perception (Smith, 2015), depression (Da Silva et al., 2020), stress (Da Silva et al., 2020), attention (Ahmed and Mohamed, 2011), cognitive flexibility (Da Silva et al., 2020), and response inhibition (Verret et al., 2012). It is important that further research is done to highlight which contexts influence these outcomes within this population, both positively and negatively. For example, Barnett et al. (2011) has recommended targeting sports competence alongside PA levels and skill development because of its influence on active lifestyles of children who are TD. The complexity of an individual’s exercise experience and social context was highlighted by Ninot et al.’s (2005) adverse finding of significantly lower athletic self-perception in children with ID who trained and competed against children who are TD in swimming. Children with ID reported lower athletic self-perception despite an improvement in their own swimming times indicating comparative aspects of exercise contexts can impact a child’s self-perception, enjoyment (Ninot et al., 2005); and ultimately their engagement in exercise.

Child engagement was facilitated in some studies through parent involvement. Home exercise programs may play an important role in facilitating carryover of exercise interventions into a child’s home environment (Huang and Pang, 2010; Kane and Staples, 2016). They may also provide both children and parents with ideas and experiences of successful and sustainable exercise at home. In addition, studies of children who are TD have found that doing exercise together with their parents provides opportunities for parental role modelling of exercise (Yao and Rhodes, 2015), bonding and connecting experiences, and opportunities for children to practice their developing skills in a safe environment (Freire et al., 2019). It was disappointing how little attention was given to assessing and evaluating potential benefits of home exercise programs of the included studies. Future studies should focus on how to evaluate this aspect, beyond engagement levels.

Social ecological models of health describe the complex interplay between children, families, their communities, and society (Bronfenbrenner, 1989). Importantly, they acknowledge that children influence their parents and their environment (James et al., 1998). Adopting a social ecological approach may increase the number of studies taking a holistic approach to child outcome measures (physical, social, psychological). This approach would also increase the number of studies involving parent with interventions and seeking the child’s view of exercise interventions. Finally, social ecological models of health would likely increase the number of studies utilising exercise interventions that were also available at home or in their community. A small number of studies in this review included novel exercise interventions that were unlikely to have been available in a child’s home or community or focused upon skills that children may not be able to utilise at home; for example, using a simulated developmental horse-riding machine (Pan et al., 2017a) and interventions focussed upon table tennis skills (Pan et al., 2016). Researchers should use exercise interventions that are relevant to the child’s community context, to increase the possibility of exercise interventions having long-term positive impacts upon total PA levels and health.

### Limitations of included studies

Limitations of studies included moderate (*n* = 20, 41%) or low (*n* = 18, 37%) methodological quality of studies, which suggests high risk of bias. Design limitations, in particular small sample sizes, may have contributed to some of the null findings. Lack of a control group or utilisation of a no-intervention control group was another common limitation, whilst in contrast other studies used a waitlist design, which arguably seems a more ethically acceptable approach with this vulnerable population whilst also benefiting sample sizes. Confounding factors, such as comparing two different interventions with two different group sizes (which may have affected group interactions), were found. Additionally, the influence of the social element of the intervention or group context was often not adequately considered, with many studies (*n* = 18, 37%) making no attempt to measure impact upon other, non-physical aspects of a child’s development, either positive or negative. This limitation is underlined by some adverse findings, which may have been related to group context.

Parent and teacher voices were sought across a small number of studies but only one study, by Siu and Lo (2020), sought to gain the child’s perspective on a family rugby intervention. They found that children felt they had better self-control post intervention. The absence of children’s voices is an important gap in the research and future studies should consider ways to elicit children’s opinions of exercise interventions. Seeking child and family input into exercise interventions, prior to implementing them, via participatory approaches, would also increase the relevance of exercise interventions to target populations (Freire et al., 2022).

### Limitations of review

The review is limited by likely publication bias and inclusion of only studies published in English between years of 2000 and 2021. Also, heterogeneity and low methodological quality of most of the included studies made it difficult to compare across studies. These factors limited analyses to narrative review, increasing the risk of reviewer bias, but comprehensive tables providing details of studies and extracted data have been compiled to increase review transparency (Munn et al., 2014). In addition, absence of, or insufficient description of the exercise interventions was a limitation in some studies and may have also erroneous grouping within this systematic review.

### Implications for practice

Findings of this review suggest that group exercise intervention contexts influence children’s experiences and holistic development positively or negatively highlighting the importance of planning and evaluating the psychosocial impact of group exercise interventions upon this population. Although short-term improvements in motor skills were found following group interventions, many of the interventions did not support children to *meet* their developmental motor milestones, suggesting that longer interventions or booster sessions are required to provide children who are not meeting their developmental milestones with greater opportunities to thrive.

## Conclusion

This review found that group exercise interventions with a social element for children who are not meeting their developmental milestones, may improve more than children’s motor skills. However, current evidence is of poor quality. Few studies took a comprehensive and holistic approach to assessing children’s developmental outcomes. Thus, further robust research is needed to establish the full impact of group exercise interventions upon social, psychological, executive function outcomes and total PA levels. Future research and exercise interventions should include longitudinal intervention studies or booster sessions to ensure that children are given greater opportunities to thrive and *meet* their developmental milestones.

## Supplemental Material

Supplemental Material - Social exercise interventions for children who have complex developmental needs: A systematic review Supplemental Material for Social exercise interventions for children who have complex developmental needs: A systematic review by Kate Freire, Rod Pope, Isabella Size, Kristen Andrews, Emma Fitz-Gerald and Tricia Bowman in Journal of Child Health Care
