# Supplementary material for: Social exercise interventions for children who have complex developmental needs: A systematic review
Source: J Child Health Care. 2023 Jul 20;29(1):245–87. doi: 10.1177/13674935231190984 (PMC11874478; doi:10.1177/13674935231190984)
Supplement: Supplemental Material - Social exercise interventions for children who have complex developmental needs: A systematic review [file sj-pdf-1-chc-10.1177_13674935231190984.pdf]

Search strategy - Social PA SR Search strategy - Supplemental material for Social physical activity interventions for children who have complex developmental needs: a systematic review; Search strategy – Supplemental material, Social PA SR Search strategy, for Social physical activity interventions for children who have complex developmental needs: a systematic review by Freire, Pope, Size, Andrews, Fitz-Gerald, & Bowman in Journal of Child Health Care.

Methodological Quality Screening of Included Studies using MMAT – MMAT screen Social PA SR Jan 23 - Supplemental material for Social physical activity interventions for children who have complex developmental needs: a systematic review; Methodological Quality Screening of Included Studies using MMAT – Supplemental material, Social PA SR Search strategy, for Social physical activity interventions for children who have complex developmental needs: a systematic review by Freire, Pope, Size, Andrews, Fitz-Gerald, & Bowman in Journal of Child Health Care.

For Peer Review

Summary table of outcome measures utilised across included studies – Social PA SR summary outcome measures - Supplemental material for Social physical activity interventions for children who have complex developmental needs: a systematic review; summary of outcome measures utilised across included studies - Supplemental material, Social PA SR summary outcome measures, for Social physical activity interventions for children who have complex developmental needs: a systematic review by Freire, Pope, Size, Andrews, Fitz-Gerald, & Bowman in Journal of Child Health Care.

Synthesis of schedules of PA Interventions - Social PA SR Synthesis of schedules of PA Interventions - Supplemental material for Social physical activity interventions for children who have complex developmental needs: a systematic review; Synthesis of schedules of PA Interventions - Supplemental material, Social PA SR Synthesis of schedules of PA Interventions - for Social physical activity interventions for children who have complex developmental needs: a systematic review by Freire, Pope, Size, Andrews, Fitz-Gerald, & Bowman in Journal of Child Health Care.

Search Strategy

Academic Search Complete – Ebscohost	CINAHL – Ebscohost	Emcare – Ovid	Proquest Theses and Dissertations	MEDLINE - Pubmed
(MJ "Physical Activity") OR (MH "Exercise") OR (MJ "sports") OR (AB "Chores") OR (AB "active transport") OR (AB "recreation*") OR (AB "leisure")<br>AND<br>"developmental* vulnerab*" OR "social competence" OR "emotional maturity" OR "communication disorder" OR "well-being" OR "motor skills"<br>AND<br>intervention OR "strateg" OR program*<br>AND<br>child* selected "<br>Apply equivalent subjects" option limited to 2000-2020, Academic journals, dissertations English language	(MJ "Physical Activity") OR (MH "Exercise") OR (MJ "sports") OR (AB "Chores") OR (AB "active transport") OR (AB "recreation*") OR (AB "leisure*")<br>AND<br>"developmentally vulnerable" OR "social competence" OR "emotional maturity" OR "communication disorder" OR "well-being" OR "motor Skills"<br>AND<br>intervention OR "strateg" OR program*<br>Apply "equivalent subjects"<br>Limit to 2000 – 2020 Limited to "all child"	AB ("Physical Activity" OR "Exercise" OR "sport*" OR "Chores" OR "active transport" OR "recreation*" OR "leisure")<br>AND<br>AB ("developmental* vulnerab*" OR "social competence" OR "emotional maturity" OR "communication disorder" OR "well-being" OR "motor Skills")<br>AND<br>AB (intervention OR "strateg" OR program*)<br>AND<br>SU (child*)<br>Limited to human and English Language	AB ("Physical Activity" OR "Exercise" OR "sports" OR "Chores" OR "active transport" OR "recreation*" OR "leisure")<br>AND<br>AB (developmental* vulnerab* OR "social competence" OR "emotional maturity" OR "communication disorder" OR "well-being" OR "motor Skills")<br>AND<br>AB (intervention OR "strateg" OR program*)<br>AND<br>AB (child*)<br>Limited to English Language	[Title/Abstract] ("Physical Activity" OR "Exercise" OR "sport*" OR "Chores" OR "active transport" OR "recreation*" OR "leisure")<br>AND<br>[Title/Abstract] developmentally vulnerable" OR "social competence" OR "emotional maturity" OR "communication disorder" OR "well-being" OR "motor Skills"<br>AND<br>[Title/Abstract] intervention OR "strateg" OR program*<br>AND<br>Child*<br>Limited to human and English Language

For Peer Review

Methodological Quality Screening of Included Studies Using MMAT

Authors, date	Study design	MMAT rating	Comment
Fong et al., 2012	RCT	*****	All criteria met
Hung & Pang, 2010	RCT	*****	All criteria met
Tsai et al., 2012	RCT	****	C2.1 not met
Pan et al., 2016	RCT	***	C2.1, 2.4 not met
Pan et al., 2017b	RCT	***	C2.1, 2.4 not met
Sullivan, 2017	RCT	***	C2.1, 2.4 not met
Valentini et al., 2017	RCT	***	C2.1, 2.4 not met
Favazza et al., 2013	RCT	**	C2.1, 2.2, 2.4 not met
Memarmoghaddam et al., 2016	RCT	**	C2.1, 2.2, 2.4 not met
Peens et al., 2008	RCT	**	C2.1, 2.2, 2.4 not met
Pless, 2001 Study 2	RCT	**	C2.1, 2.4, 2.5 not met
Tsiotra, 2010, Study 3	RCT	**	C2.1, 2.4, 2.5 not met
Ahmed & Mohamed, 2011	RCT	*	C2.1, 2.3, 2.4, 2.5 not met
Dickinson & Place, 2016	RCT	*	C2.1, 2.2, 2.4, 2.5 not met
Najafabadi et al., 2018	RCT	*	C2.1, 2.2, 2.3, 2.5 not met
Őzer et al., 2012	RCT	*	C2.2, 2.3, 2.4, 2.5 not met
Regaieg et al., 2020	RCT	*	C2.1, 2.3, 2.4, 2.5 not met
Ziereis & Jansen, 2015	RCT	*	C2.1, 2.3, 2.4, 2.5 not met
Da Silva et al., 2020	RCT		No criteria met
Lee et al., 2015	RCT		No criteria met
Angeli et al., 2019	NRCT	*****	All criteria met
Apache, 2005	NRCT	*****	All criteria met
Bremer et al., 2015	NRCT	*****	All criteria met
Chou & Huang, 2017	NRCT	*****	All criteria met
Ferguson et al., 2013	NRCT	*****	All criteria met
Ferguson et al., 2015	NRCT	*****	All criteria met
O'Connor et al., 2014	NRCT	*****	All criteria met
Pan et al., 2017a	NRCT	*****	All criteria met
Smith, 2015	NRCT	*****	All criteria met
Chang et al., 2014	NRCT	****	C3.1 not met
Işık & Zorba, 2020	NRCT	****	C3.5 not met
Kane & Staples, 2016	NRCT	****	C3.5 not met
Ninot et al., 2005	NRCT	****	C3.3 not met
Rafie et al., 2017	NRCT	****	C3.4 not met
Smith et al., 2013	NRCT	****	C3.4 not met
Tsai, 2009	NRCT	****	C3.3 not met
Verret et al., 2012	NRCT	****	C3.5 not met
Wing-Chung et al., 2020	NRCT	****	C3.5 not met
Bo et al., 2019	NRCT	***	C3.1, 3.4 not met
Caçola et al., 2016	NRCT	***	C3.1, 3.5 not met
Golubović et al., 2014	NRCT	***	C3.4, 3.5 not met
Ketcheson et al., 2017	NRCT	***	C3.3, 3.5 not met
Oreskovic et al., 2020	NRCT	***	C3.4, 3.5 not met

For Peer Review

Authors, date	Study design	MMAT rating	Comment
Ketcheson et al., 2021	NRCT	**	C3.3, 3.4, 3.5 not met
Kosari et al., 2013	NRCT	**	C3.3, 3.4, 3.5 not met
Rubin et al., 2018	NRCT	**	C3.2, 3.4, 3.5 not met
Ekins et al., 2019	NRCT	*	C3.1, 3.3, 3.4, 3.5 not met
Bremer & Lloyd, 2016	MM	****	C5.5 not met
Siu & Lo, 2020	MM	*	C5.2, 5.3, 5.4, 5.5 not met

RCT: randomised controlled trial, NRCT non-randomised controlled trial, MM mixed methods.

Note: Studies listed by MMAT score in study design and alphabetical order.

Mixed Methods Appraisal Tool (MMAT), version 2018. Hong et al., 2018.

For Peer Review

Summary table of outcome measures utilised across included studies

**Physical outcome measures**
4 corporal coordination tests for children (Schilling and Kiphard, 1976)
20-meter shuttle run test, also known as bleep test (Ramsbottom et al., 1988)
Backward Obstacle Course Test (Bala and Popović, 2007)
Basic Motor Ability Test (Arnheim and Sinclair, 1979)
Body Mass Index
Bruce treadmill protocol (Wessel, Strasburger and Mitchell, 2001)
Bruininks-Oseretsky Test of Motor Proficiency (Bruininks and Bruininks 2005)
Children Activity Scale (Rosenblum, 2006)
Children's Self-perceptions of Adequacy in and Predilection for Physical Activity Scale (Hay, 1992)
Chin ups
Developmental Coordination Disorder Questionnaire (Wilson et al., 2000)
Eurofit physical fitness battery (Council of Europe, 1993)
Hand tapping test (Bala and Popović, 2007)
Heart rate (resting and maximal)
Height
Home exercise compliance log
German Motor Skill Test (Bös et al., 2009)
Grip strength using handheld dynamometer
Motor timing task (Zelaznik, Spencer and Ivry, 2008)
Movement Assessment Battery for Children (Henderson et al., 2007)
Muscle Power Sprint Test (Verschuren et al., 2007)
Parent rating on Likert scale to the following questions: "How much did your child improve in sports skills?" and "How much did your child improve in good sportsmanship?"
Partial curl ups
Peabody Developmental Motor Scale (Folio and Fewell, 2000)
Pedometers
Physical activity participation questionnaire
Push ups
Sensory organization test (Nashner, 1997)
Sit and reach test
Skill accuracy tasks: dribbling football, kicking, trapping. Throwing balls, catching, hitting.
Sport knowledge and performance tasks
Standing long jump (Bala and Popović, 2007)
Step test
Test of Gross Motor Development (Ulrich, 2016)
The Behavior Rating Scale (Fisher and Newby, 1991)
Timed sit ups
Timed half mile run
Timed swim test (50m breaststroke with diving start)

For Peer Review

Total daily physical activity levels with accelerometers
Unilateral stance test
Verbal recall checklist of sporting rules
Vineland Teacher Rating Form (Sparrow, Cicchetti and Balla, 2005)
Weight
**Social and behavioural outcome measures**
Autism Treatment Evaluation Checklist (Memari et al., 2013)
Behavior questionnaire for developmental disabilities (used in Ekins et al., 2019 but no reference provided)
Behavioural video coding of free play using Social Behaviour Codes from Hauck et al. (1995)
Child Behaviour Checklist (Achenbach, 1991)
Children’s Assessment of Participation and Enjoyment (King et al, 2006)
Connors Rating Scale (Conners, 2008)
Developmental Behavior Checklist (Dekker et al., 2002)
Family Adaption and Cohesion Evaluation Scales (Olson, Gorall and Tiesel, 2006)
Friendship Activity Scale (Siperstein and Bak, 1985)
Gilliam Autism Rating Scale (Gilliam, 2006)
Heidelberg Competency Inventory (used in Ekins et al., 2019 but no reference provided)
Parent rating on Likert scale to the following question: “How much did your child improve in good sportsmanship?”
Parent-Child Interaction Questionnaire (Lange et al., 2002)
Playground Observation of Peer Engagement (Frankel et al., 2011)
Preferences for Activities of Children (King et al, 2006)
Social Skills Improvement System (Gresham and Elliott, 2008).
Strengths and Difficulties Questionnaire (Elander and Rutter, 1996)
The Behavior Rating Scale (Fisher and Newby, 1991)
Strengths and Weakness of ADHD Symptoms and Normal Behavior (Swanson et al., 2012)
Vineland Teacher Rating Form (Sparrow, Cicchetti and Balla, 2005)
Post intervention teacher and parent survey of perceived benefits of intervention
**Psychological and mental health outcome measures**
Beck Inventory for Anxiety (Cunha, 2001)
Body-Mind-Spirit Well-Being Inventory (parenting stress) (Ng et al., 2005)
Child Anxiety Scale (Gillis, 1980)
Child depression inventory (Cruvinel et al., 2008)
Children and Youth Physical Self-Perception Profile (Fox and Corbin, 1989)
Children’s Self-Perceptions of Adequacy in and Predilection for Physical Activity Scale (Hay, 1992)
Modified Mood Rating Scale (Miodrag, Lense and Dykens, 2012)
Perceived Stress Scale (Cohen et al., 1983)
Self-perception profile for children (Harter, 2012)
Spence’s Child Anxiety Scale (Spence, 1998)
Strengths and Difficulties Questionnaire (Goodman and Goodman, 2009)

For Peer Review

Tennessee Self-concept Scale (Fitts and Warren, 1996)
The Adjective Checklist (Siperstein, 1980)
The Self-Perception Profile (Pierrehumbert et al., 1987)
**Executive function outcome measures**
Behaviour Rating Inventory of Executive Function (Gioia et al, 2000)
Corsi block tapping test (Pagulayan et al., 2006)
Go/no go task (Gordon and Caramazza, 1982)
HAWIK IV (Petermann and Petermann, 2010)
Mazes sub-test from Wechsler Pre-school and Primary Scale of Intelligence (Wechsler, 1989)
NEPSY-II subtests executive function (Korkman et al., 2007)
Numbers Reversed subtest from Woodcock-Johnson III Tests of Cognitive Abilities (Woodcock, McGrew and Mathur, 2001)
Posner Paradigm Test (Posner, Rothbart and Sheese, 2007)
Shape School (Epsy, 1997)
Stroop test (Golden, Freshwater and Golden, 2003)
Test of Everyday Attention for Children (Manly et al., 1999)
The Behavior Rating Scale (Fisher and Newby, 1991)
The Determination Test (Shmygalev et al., 2011).
The TAC Cancellation Attention Test (Montiel and Seabra, 2012)
The Visual Pursuit Test of the Vienna Test System (Schmid et al., 2005)
Visuospatial Attention Orienting Task (Tsai et al., 2010)
Wide Range Assessment of Memory and Learning (Sheslow and Adams, 2003)
Wisconsin Card Sorting Test (Heaton et al., 1993)
Word test (Golden, Freshwater and Golden, 2003)
**Other outcome measures**
Parent or guardian satisfaction surveys
Rating scales of child and parent perception of child attainment of their goal

N.B. Some measures are listed in more than one domain because they cover several domains. However, some authors in the SR used subscales which did not cover all the domains. Different versions of outcome measure were used in different studies included in the review, so versions have not been stipulated in this appendix.

References

Achenbach TM 1991 *Manual for the child behavior checklist 4–18 and 1991 profile.* Burlington: University of Vermont.

Arnheim D and Sinclair WA (1979) The basic motor ability tests revised. In D. Arnheim, & S. Sinclair (Eds.), The clumsy child (2nd ed., pp. 119–144). St Louis: Mosby.

For Peer Review

Bala G and Popović B (2007) Motoricˇke sposobnosti predškolske dece. In: *Antropološke karakteristike i sposobnosti predškolske dece* (eds Bala, U. & Ured, G.), pp. 101–150. Fakultet sporta i fizicˇkog vaspitanja, Novi Sad.

Bös K, Schlenker L, Büsch D et al (2009) Deutscher Motorik-Test 6-18 (DMT 6-18) [German motor ability test 6-18]. Hamburg: Czwalina.

Bruininks RH and Bruininks B (2005) Bruininks-Oseretsky test of motor proficiency (2nd ed.). Minneapolis, MN: NCS Pearson.

Cohen S, Karmack T, and Mermelsteinn R (1983) A global measure of perceived stress. *Journal of Health Social Behavior* 24: 385–396.

Conners CK (2008) *Conner's Rating Scales for Attention Deficit Hyperactivity Disorder.* 3rd ed. Multi Health System, North Tonawanda, New York.

Council of Europe (1993) *Eurofit Tests of Physical Fitness.* Council of Europe, Strasbourg, France, 2nd ed.

Cruvinel M, Boruchovith E and Santos AA (2008) Inventário de depressão infantil (CDI): análise dos parâmetros psicométricos. *Fractal Revista De Psicologia* 2:473–490.

Cunha JA (2001) *Manual da Versão em Português das Escalas Beck*. São Paulo: Casa do Psicólogo; p. 171.

Dekker MC, Nunn RJ, Einfeld SE et al (2002) Assessing emotional and behavioral problems in children with intellectual disability. Revisiting the factor structure of the Developmental Behaviour Checklist. *Journal of Autism and Developmental Disorders* 32(6): 601-610.

Elander J and Rutter M (1996) Use and development of the Rutter Parents' and Teachers' Scales. *International Journal of Methods in Psychiatric Research* 6(2): 63-78.

Espy KA (1997) The Shape School: Assessing executive function in preschool children. *Developmental Neuropsychology* 13(4): 495-499.

Fisher M and Newby RF (1991). Assessment of stimulant response in ADHD children using a refined multimethod clinical protocol (special issue on child psychopharmacology). *Journal of Clinical Child Psychology* 20: 232-44.

Fitts WH and Warren WL (1996) *Tennessee Self-Concept Scale*, 2nd ed. Western Psychological Services, Los Angeles, CA, USA.

Folio MR and Fewell RR (2000) *Peabody Developmental Motor Scales-2*. Austin, TX: Pro-Ed.

For Peer Review

Fox KR and Corbin CB (1989) The physical self-perception profile: development and preliminary validation. Journal of Sport and Exercise Psychology 11(4):408-430.

Frankel FD, Gorospe CM, Chang Y et al. (2011) Mothers' reports of play dates and observation of school playground behavior of children having high-functioning autism spectrum disorders. *Journal of Child Psychology and Psychiatry* 52(5): 571–579.

Gilliam JE (2006) GARS-2: *Gilliam autism rating scale:* Pro-ed.

Gillis JS (1980) *Child Anxiety Scale*. Western Psychological Services, Los Angeles, CA, USA.

Gioia GA, Isquith PK, Guy SC et al. (2000). Test review. Behavior rating inventory of executive function. *Child Neuropsychology* 6(3): 235–238.

Golden CJ Freshwater SM and Golden Z (2003) *Stroop color and word test: children's version for ages 5–14.* Wood Dale, Illinois: Stoelting Co.

Goodman A and Goodman R (2009) Strengths and difficulties questionnaire as a dimensional measure of child mental health. *Journal of the American Academy of Child and Adolescent Psychiatry* 48(4):400-403.

Gordon B and Caramazza A (1982) Lexical decision for open- and closed-class words: Failure to replicate differential frequency sensitivity. *Brain and Language* 15(1):143–160.

Gresham FM and Elliott SN (2008) *Social Skills Improvement System*. Minneapolis, MN: Pearson Education.

Harter S (2012) Self-perception profile for children: Manual and questionnaires (revision of the self-perception profile for children, 1985). Denver: University of Denver.

Hauck M, Fein D, Waterhouse L et al. (1995) Social initiations by autistic children to adults and other children. *Journal of Autism and Developmental Disorders* 25(6): 579–595.

Hay J (1992) Adequacy in and predilection for physical activity in children. *Clinical Journal of Sports Medicine* 2(3):192-201.

Heaton RK, Chelune GJ, Talley JL et al. (1993) *Wisconsin Card Sorting Test Manual: Revised and Expanded*. Lutz, FL: Psychological Assessment Resources.

Henderson SE, Sugden DA and Barnett AL (2007) *Movement Assessment Battery for Children-2*. London, UK: Pearson Education.

For Peer Review

King GA, Law M, King S et al. (2006) Measuring children's participation in recreation and leisure activities: construct validation of the CAPE and PAC. *Child: care, health & development* 33(1):28-39.

Korkman M, Kemp SL and Kirk U (2001) Effects of age on neurocognitive measures of children ages 5 to 12: A cross-sectional study on 800 children from the United States. *Developmental Neuropsychology* 20(1): 331-354.

Lange A, Evers A, H. Jansen H et al. (2002) PACHIQ-R: the parent-child interaction questionnaire - revised. *Family Process* 41(4): 709–722.

Manly T, Robertson IH, Anderson V et al. (1999) *TEA-Ch: The test of everyday attention.* Bury St-Edmunds, England: Thames Valley Test Company Limited.

Memari AH, Shayestehfar M, Mirfazeli FS et al (2013) Cross-cultural adaptation, reliability, and validity of the autism treatment evaluation checklist in Persian. *Iran Journal of Pediatr*ics 23:269-275.

Miodrag N, Lense MD and Dykens EM (2013) A pilot study of a mindfulness intervention for individuals with Williams syndrome: Physiological outcomes. *Mindfulness* 4(2): 137-147.

Montiel JM and Seabra AG (2012) Teste De Atenção Por Cancelamento. In: Seabra A, Dias N, editors. Orgs. *Avaliação Neuropsicológica Cognitiva: atenção e Funções Executivas*. São Paulo: Memnon; p. 66.

Nashner LM (1997. Computerized dynamic posturography. In G. P. Jacobson, C. W. Newman, & J. M. Kartush (Eds.), *Handbook of balance function and testing* (pp.261–307). St. Louis Mosby.

Ng SM, Yau JK, Chan CL et al. (2005) The measurement of body-mind-spirit well-being: toward multidimensionality and transcultural applicability. *Social Work in Health Care* 41(1): 33–52.

Olson DH, Gorall DM and Tiesel (2006) *FACES-IV Package:Administration*, Life Innovations, Minneapolis, Minn, USA.

Pagulayan K Busch R, Medina K et al. (2006) Developmental normative data for the corsi block-tapping task. *Journal of Clinical and Experimental Neuropsychology* 28(6): 1043–1052.

Petermann F and Petermann U (2010) *HAWIK-IV*. Hamburg-Wechsler-Intelligenztest fu¨r Kinder – IV. Manual 3, erga¨nzte Auflage. Bern: Huber.

For Peer Review

Pierrehumbert B, Plancherel B and Jankech-Caretta C (1987) Image de soi et perception des compétences propreschez l'enfant. *Revue de Psychologie Appliquée* 4: 359–77.

Posner MI and Cohen Y (1984) *Components of visual orienting*. In H Bauma & DG Bouwhis (Eds.), Attention and performance (pp. 531–556). Hillsdale, NJ: Erlbaum.

Ramsbottom et al. (1988) A progressive shuttle run test to estimate maximal oxygen uptake. *British Journal of Sports Medicine* 22: 141-5.

Rosenblum S (2006) The development and standardization of the Children Activity Scales (ChAS-P/T) for the early identification of children with Developmental Coordination Disorders. *Child: care, health & development* 32(6): 619-632.

Schilling F and Kiphard EJ (1976) The Body Coordination Test. *Journal of Physical Education and Recreation* 47(4): 37-39.

Schmid G, Sauter C, Stepansky R et al. (2005) No influence on selected parameters of human visual perception of 1970 MHz UMTS-like exposure. *Bioelectromagnetics* 26(4): 243-350.

Sheslow D and Adams W (2003) *Wide range assessment of memory and learning* (2nd ed.). Los Angeles, CA: Western Psychological Services.

Shmygalev S, Damm M, Weckbecker K et al (2011) The impact of long-term maintenance treatment with buprenorphine on complex psychomotor and cognitive function. *Drug and Alcohol Dependence* 117(2):190- 197.

Siperstein GN (1980) Instruments for measuring children's attitudes toward the handicapped. Unpublished manuscript. University of Massachusetts at Boston.

Siperstein GN and Bak JJ (1985) Effects of social behavior on children's attitudes toward their mildly and moderately mentally retarded peers. *American Journal Mental Deficiency* 90(3): 319–327.

Sparrow SS, Cicchetti DV and Balla DA (2005) *Vineland Adaptive Behavior Scales-2*. Bloomington, MN: Pearson Education.

Spence SH (1998) A measure of anxiety symptoms among children. *Behaviour Research and Therapy* 36(5):545-566.

Swanson S, Schuck M, Mann C et al (2012) Categorical and dimensional definitions and evaluations of symptoms of ADHD: history of the SNAP and the SWAN Rating Scales. *International Journal Educational Psychology* 10(1): 51–70.

For Peer Review

Tsai CL, Pan CY, Chang YK et al. (2010). Deficits of visuospatial attention with reflexive orienting induced by eye-gazed cue in children with developmental coordination disorder on lower extremities: An event-related potential study. *Research in Developmental Disabilities* 31(3): 642–655.

Ulrich DA (2016) *Test of gross motor development (3rd ed.)*. Austin: Pro-ed publisher.

Verschuren O, Takken T, Ketelaar M et al. (2007) Reliability for running tests for measuring agility and anaerobic muscle power in children and adolescents with cerebral palsy. *Pediatric Physical Therapy* 19: 108–115.

Wechsler D (1989) *Wechsler Preschool and Primary Scale of Intelligence–Revised*. San Antonio, TX: Psychological Corporation.

Wessel HA, Strasburger JF and Mitchell BM (2001) New standards for the Bruce treadmill protocol in children and adolescents. *Pediatric Exercise Science* 13(4): 392-401.

Wilson BN, Kaplan BJ, Crawford SG et al (2000) Reliability and validity of a parent questionnaire on childhood motor skills. *American Journal of Occupational Therapy* 54:484-493.

Woodcock RW, McGrew KS and Mather N (2001) *Woodcock-Johnson III tests of cognitive abilities*. Itasca, IL: Riverside.

Zelaznik HN, Spencer RMC and Ivry RB (2008) Behavioral analysis of human movement timing. In S. Grondin (Ed.), *Psychology of time* (pp. 233-260). Bingley, UK: Emerald Group.

For Peer Review

Synthesis of schedules of Exercise Interventions

Groups of studies	Mean duration of intervention (weeks)	Range: duration of intervention (weeks)	Median duration of PA session (mins)	Range: duration of PA session (mins)	Mean frequency of sessions per week	Range: frequency of sessions per week
**Group 1: Studies which incorporated social element (n = 24)**	9.5	1-24	60 (n = 21)	30 – 240 (n = 21)	2.9	1 - 5
**Group 2: Studies where social element was incidental (n = 25)**	15.9 (n = 22)	2 - 139 (n = 22)	45.5 (n = 22)	15 – 240 (n = 22)	2.4 (n = 21)	1 -5 (n = 21)

Note: not all studies provided information on their intervention schedule.

For Peer Review
